# Epicardial Adipose Tissue in Arrhythmogenic Cardiomyopathy

**DOI:** 10.3390/biology14030278

**Published:** 2025-03-08

**Authors:** Davide Lapolla, Luca Canovi, Maria Letizia Berloni, Veronica Amantea, Cristina Balla, Federico Marchini, Evelina Faragasso, Matteo Bertini, Elisabetta Tonet

**Affiliations:** 1 Cardiology Unit, Azienda Ospedaliero Universitaria of Ferrara, 44124 FE, Italy; 2 Department of Cardiology and Cardiac Surgery, Cardiovascular Imaging, GVM Care&Research, 42121 Reggio Emilia, Italy

**Keywords:** epicardial adipose tissue, cardiac magnetic resonance, arrhythmogenic cardiomyopathy

## Abstract

Arrhythmogenic cardiomyopathy (ACM) is an inherited heart disease characterized by fibrofatty replacement of the ventricular myocardium, with an estimated prevalence of 1:5000 people in the general population. Sudden cardiac death is the first manifestation of this disease in 16–23% of patients with ACM. Fibrofatty infiltration can be identified with noninvasive cardiac magnetic resonance. Studies of epicardial fat deposits have suggested pathogenic roles of epicardial fats in mediating cardiac diseases and arrhythmias. Although myocardial fat infiltration has been well described in ACM, changes in epicardial fat deposits with this disease have not been well investigated. Our study shows that patients with ACM have a higher amount of EAT compared to controls. Additionally, the EAT amount seems to increase with the evolution of the disease.

## Simple Summary

Arrhythmogenic cardiomyopathy (ACM) is characterized by fibrofatty replacement of the ventricular myocardium. Fibrofatty infiltration can be identified with noninvasive cardiac magnetic resonance. Studies of epicardial fat deposits have suggested pathogenic roles of epicardial fats in mediating cardiac diseases and arrhythmias. Although myocardial fat infiltration has been well described in ACM, changes in epicardial fat deposits with this disease have not been well investigated. Our study shows that patients with ACM have a higher amount of EAT compared to controls. Additionally, the EAT amount seems to increase with the evolution of the disease.

## 1. Introduction

Arrhythmogenic cardiomyopathy (ACM) is a disease characterized by a progressive myocardial fibrofatty replacement of the right (RV) and left ventricular (LV) myocardium [1]. It can either involve only one ventricle or be biventricular. The amount of fibrosis and/or adipose tissue is extremely variable; this could be related to pathogenetic variants, but it remains unclear so far [2,3]. The real incidence and prevalence of this disease are unknown, but ACM is a well-recognized cause of sudden death in adolescents and young people [4]. Fibrofatty infiltration is a typical feature of ACM and can be identified with cardiac magnetic resonance (CMR) [5,6,7]. This infiltration is responsible for ventricular arrhythmias. Whether epicardial adipose tissue is also involved in inflammation and the arrhythmogenic substrate of ACM hearts is unknown. As a matter of fact, some studies have shown that epicardial adipose tissue (EAT) thickness positively correlates with the ventricular ectopy burden, QT interval prolongation (>450 ms), and ventricular tachycardia (VT) in the context of heart failure and with VT recurrence post ablation [8,9,10]. Furthermore, the inhomogeneous distribution of EAT around the myocardium may result in heterogeneity in action potential duration, thereby generating an arrhythmogenic substrate enabling re-entry. Cytokines that are known to be actively secreted by EAT, such as IL-1b, TNF-a, and IL-6, have been shown to alter ion channel function and may mediate part of the increased arrhythmogenicity associated with increasing adiposity. Cardiac mesenchymal stromal cells from human hearts with ACM and the plakophilin-2 pathogenetic variant are abnormally adipogenic and have been suggested to be the source of pathologic fatty formation in ACM hearts. Because the human epicardium is rich in cells with mesenchymal characteristics, patients with ACM may also have a higher amount of EAT [11]. The hypothesis that EAT could have a role in the pathogenesis and evolution of ACM could support adding EAT evaluation to standardized CMR in patients suffering from ACM. Knowing that EAT has a role in ACM could lead to improvements in the management and risk stratification of these patients.

The purpose of this study was to analyze EAT in patients with ACM and compare the EAT thickness measured by CMR in these patients with that in the control group without known cardiovascular disease.

## 2. Materials and Methods

### 2.1. Population

We analyzed 36 consecutive patients with ACM who underwent CMR between June 2014 and October 2022. The diagnosis of ACM was based on fulfilling the revised Task Force Criteria published by Marcus et al. in 2010 or the recently published Padua criteria, which offer an updated iteration to include LV involvement. [4] Clinical data were collected for all patients, including cardiovascular risk factors, clinical history, implantable cardioverter–defibrillator implantation, EKG Holter evaluation, transthoracic echocardiography, CMR, pharmacological treatment, genetic testing and family screening. A control group was selected from consecutive patients without structural heart disease undergoing CMR between January 2022 and October 2022. Subsequently, matching was performed for age, sex, and body mass index between the two groups.

### 2.2. Quantification of Fat

**Figure 1:**
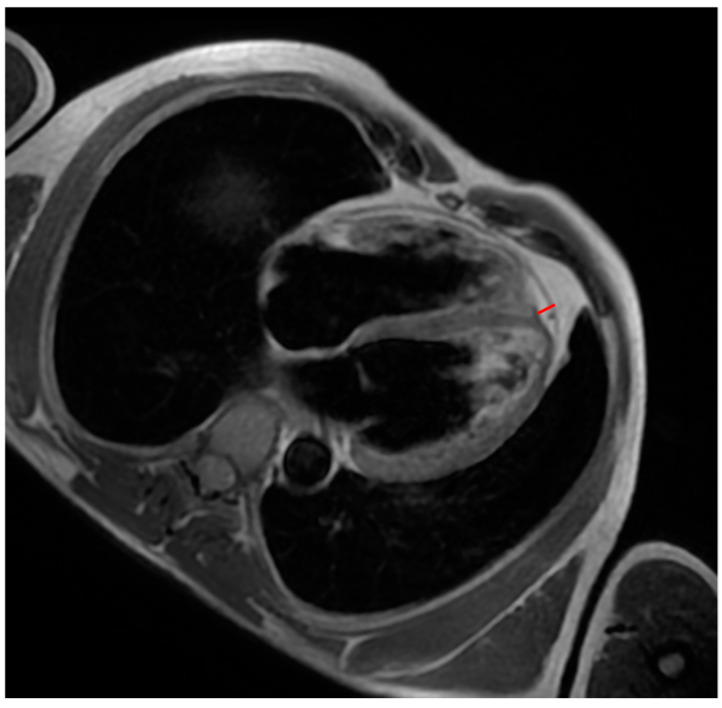
A patient with ACM. Black-blood T1-weighted 4-chamber image shows an example of measuring interventricular groove EAT (red line).

All patients underwent CMR on a 1.5-T whole-body scanner. The CMR protocol included the following: (a) breath-holding steady-state free precession (SSFP) sequences (short axis covering the entire LV and 2-, 3-, 4-chamber long-axis views, RV inflow, sagittal RV outflow tract) for functional analysis and quantification of ventricular volumes and ejection fraction; (b) black-blood T1-weighted imaging to assess fat infiltration; (c) late gadolinium enhancement (LGE) imaging according to our protocol; and (d) T1 mapping sequences. Image processing was performed by two observers (F.M./E.T.) with more than 2 years of cardiac imaging analysis experience. For each CMR image, contours were manually created to segment fat in the epicardium. EAT thickness was measured in the best sequence (SSFP or black-blood T1-weighted sequence) (Figure 1), according to a previously published method [12].

It was assessed in the following sites: right atrial EAT, LV and RV free wall EAT, and LV and RV EAT in the atrioventricular and interventricular grooves. Because of a very poor distribution of EAT along the left atrium and difficulties in its visualization by CMR, EAT measuring in this site was excluded.

### 2.3. Statistical Analysis

A statistical analysis was performed using SPSS, IBM 2024. Continuous variables were tested for normal distribution with the Kolmogorov–Smirnov test and a visual estimate of the Q-Q plot. Normally distributed variables were presented as mean ± SD and compared by t test and one-way ANOVA. Otherwise, the median [inter-quartile range], Mann–Whitney U, and Kruskal–Wallis tests were used. Categorical variables were summarized in terms of absolute and relative frequencies (percentages) and compared using the χ2 test.

## 3. Results

The study included 36 patients with ACM and 22 patients in the control group. The clinical and demographic characteristics of the patients are presented in Table 1.

By study design, there were no significant differences in age, sex, or body mass index between the patients with ACM and the control group. In addition, none of patients enrolled had comorbidities or other cardiovascular history. In the patients with ACM, we observed left ventricle involvement in 10 patients (30% of total), mid-wall LGE in 78% of cases, and adipose infiltration in 48%. Genetic analysis showed that 7 out of the 33 patients (19%) had pathogenetic variants of known ACM-associated genes, including 3 (42%) in *PKP2* (OMIM 602861), 1 (14%) in *DSG2* (OMIM 125671), 1 (14%) in *DSP* (OMIM 125647), and 2 (28%) in *SCN5A* (OMIM 600163). In addition, 18 patients (54%) had implantable cardioverter–defibrillator (ICD) for primary or secondary prevention (Table 2).

### Comparison of Epicardial Fat Between the Two Groups

**Figure 2:**
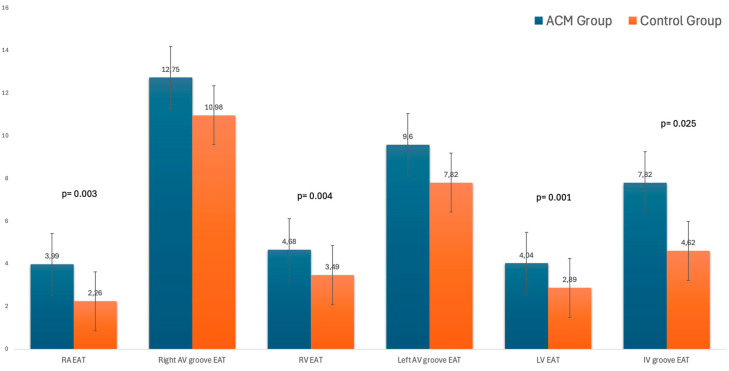
EAT values in the two groups.

The EAT was higher in the patients with ACM than in the control group (Figure 2).

In particular, the regional EAT evaluation showed that the EAT was greater in the patients with ACM than in the control group in the following sites: right atrium free wall (3.99 ± 1.1 vs. 2.26 ± 1, *p* = 0.003), right ventricular free wall (4.68 ± 1.4 vs. 3.49 ± 1.4, *p* = 0.004), left ventricular free wall (4.04 ± 1.5 vs. 2.89 ± 0.83, *p* = 0.001) for patients with LV involvement, and interventricular groove (mean value 7.82 ± 2.6 vs. 4.62 ± 2, *p* = 0.025). Meanwhile, there was no significant difference in the right and left atrioventricular grooves. During follow-up, no death and/or hospitalization were recorded, but seven patients suffered from VT at the 5-year follow-up; no differences in the EAT volume at the baseline CMR were detected. No difference was found between the three phenotypes of ACM (right/left/biventricular).

**Figure 3:**
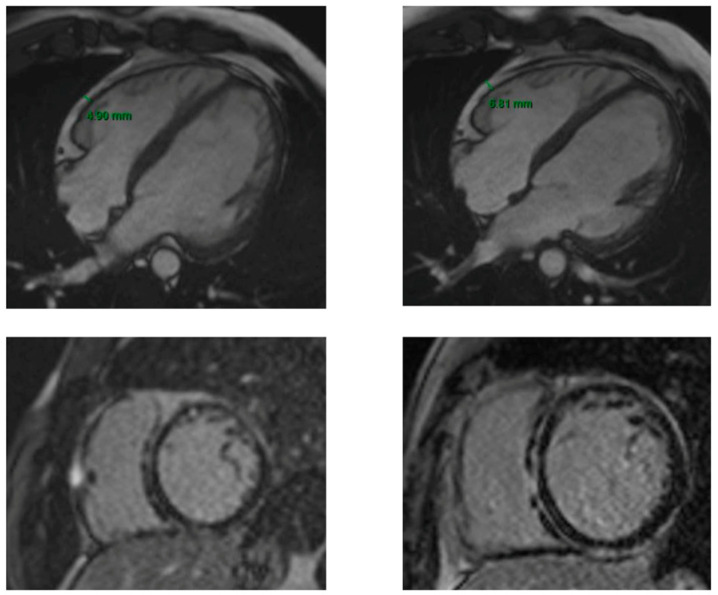
First and 2-year follow-up CMR in a patient with ACM. The two upper images are SSFP 4 chamber sequences showing the EAT measure at the baseline CMR (on the left) and at the 2-year follow-up CMR (on the right). The two lower images are LGE sequences at the baseline CMR (on the left) and at the 2-year follow-up CMR (on the right). The increase in EAT at the 2-year follow-up corresponds to a greater severity of the disease, highlighted by a more extensive LGE (ring-like distribution on the LV and RV free wall involvement).

The EAT was slightly increased in the patients with ACM (16%) at the median timing of follow-up of 2 years and correlated with LGE extension and, consequently, severity of the disease (Figure 3).

## 4. Discussion

The observation of increased EAT in patients with ACM compared to healthy individuals raises intriguing questions about its role in disease progression. Epicardial fat, as a metabolically active tissue, is anatomically and functionally contiguous with the myocardium, lacking a fibrous barrier [13,14]. This close proximity allows for direct paracrine interactions, wherein pro-inflammatory cytokines, adipokines, and free fatty acids secreted by EAT may contribute to local inflammation, oxidative stress, and fibrotic remodeling [13,15]. These mechanisms align with the hallmark pathophysiological changes in ACM, notably the replacement of normal myocardium with fibrofatty tissue.

Moreover, the increase in EAT may exacerbate electrical instability, both through direct myocardial infiltration and by promoting heterogeneity in conduction. This could amplify arrhythmic risk, a defining clinical feature of ACM [14].

The relationship between EAT and ACM, however, is likely bidirectional. While increased fat may drive disease progression, the genetic and molecular alterations inherent to ACM (e.g., desmosomal pathogenetic variants) could predispose affected individuals to greater fat accumulation, creating a pathogenic feedback loop [13,16,17].

Future research should aim to clarify whether interventions targeting EAT—such as weight management, anti-inflammatory therapies, or metabolic modulation—could mitigate disease progression or reduce arrhythmic events in patients with ACM. Understanding this interplay could pave the way for novel therapeutic strategies that address not only the structural and electrical consequences of ACM but also its metabolic and inflammatory drivers.

This study suffers from some limitations, the first of which is the small number of analyzed individuals; however, one has to consider that ACM is a rare disease and that we performed consecutive data collection and analysis, including gene pathogenetic variants and clinical mid-term follow-up. Secondly, we assessed EAT thickness but not volume, which could have confirmed our results and led to other interesting findings. Nevertheless, CMR sequences for EAT volume assessment are part of clinical practice; we focused on routine sequences with an easy and reproducible measure, ready to be used in all CMR centers. Finally, the CMR findings and EKG features were not analyzed because of a lack of EKG data.

## 5. Conclusions

Compared to healthy controls, patients with ACM have thicker EAT surrounding the ventricles, particularly epicardial fat surrounding the RV and LV, and the extent of fat shows correlation with disease progression.

## Figures and Tables

**Table 1:** Baseline characteristics of the two groups.

Variable	ACM Group	Control Group	*p*-Value
Age	50 ± 16	57 ± 11	0.1
Hypertension	12 (85.7%)	2 (14.3%)	0.03
Diabetes	1 (2.9%)	0 (0%)	0.6
Smoking	3 (8.8%)	2 (8.7%)	0.68
Cardiovascular history	0(0%)	0(%)	0
Lung disorders	0 (0%)	0 (0%)	0
Kidney disease	0 (0%)	0 (0%)	0
Active cancer/history of cancer	0 (0%)	0 (0%)	0
LVEF	57(51–65)	60 (57–61)	0.4
RVEF	55 (48–62)	59 (56–62)	0.07

LVEF = left ventricular ejection fraction; RVEF = right ventricular ejection fraction.

**Table 2:** Focus on ACM group.

Variable	Number of Patients
SVT at admission	6
NSVT at admission	8
ICD	18
Pathogenetic variants	7
• *SCN5A*	2
• *PKP2*	3
• *DSG2*	1
• *DSP*	1
ARVC	12
ALVC	10
ACM	14
Fat infiltration	16
Mid-wall LGE	26
VT event at 5-year follow-up	7

ACM = arrhythmogenic cardiomyopathy; ALVC = arrhythmogenic left ventricular cardiomyopathy; ARVC = arrhythmogenic right ventricular cardiomyopathy; ICD = implantable cardioverter–defibrillator; LGE = late gadolinium enhancement; NSVT = non-sustained ventricular tachycardia; and SVT = sustained ventricular tachycardia.

## Data Availability

Data underlying this article will be shared upon reasonable request to the corresponding author.

## Abbreviations

The following abbreviations are used in this manuscript:

EAT	Epicardial adipose tissue
ACM	Arrhythmogenic cardiomyopathy
CMR	Cardiac magnetic resonance
LGE	Late gadolinium enhancement
LV	Left ventricle
RV	Right ventricle
